# Radiation effects on atherosclerosis in atomic bomb survivors: a cross‐sectional study using structural equation modeling

**DOI:** 10.1007/s10654-021-00731-x

**Published:** 2021-03-19

**Authors:** Tomoki Nakamizo, John Cologne, Kismet Cordova, Michiko Yamada, Tetsuya Takahashi, Munechika Misumi, Saeko Fujiwara, Masayasu Matsumoto, Yasuki Kihara, Ayumi Hida, Waka Ohishi

**Affiliations:** 1 Department of Clinical Studies, Radiation Effects Research Foundation (RERF), Nagasaki, Japan; 2 Department of Statistics, RERF, Hiroshima, Japan; 3 Department of Clinical Studies, RERF, Hiroshima, Japan; 4 Faculty of Rehabilitation, Hiroshima International University, Hiroshima, Japan; 5 Faculty of Pharmacy, Yasuda Women’s University, Hiroshima, Japan; 6 Department of Clinical Neuroscience and Therapeutics, Hiroshima University, Hiroshima, Japan; 7 Department of Neurology, Sakai City Medical Center, Osaka, Japan; 8 Department of Cardiovascular Medicine, Hiroshima University, Hiroshima, Japan; 9 Kobe City Medical Center General Hospital, Hyogo, Japan

**Keywords:** Radiation exposure, Carotid intima-media thickness, Pulse wave analysis, Ankle brachial index, Latent variable modelings, Whole body irradiation

## Abstract

Past reports indicated that total-body irradiation at low to moderate doses could be responsible for cardiovascular disease risks, but the mechanism remains unclear. The purpose of this study was to investigate the association between radiation exposure and atherosclerosis, an underlying pathology of cardiovascular diseases, in the Japanese atomic bomb survivors. We performed a cross-sectional study measuring 14 clinical-physiological atherosclerosis indicators during clinical exams from 2010 to 2014 in 3274 participants of the Adult Health Study cohort. Multivariable analyses were performed by using a structural equation model with latent factors representing underlying atherosclerotic pathologies: (1) arterial stiffness, (2) calcification, and (3) plaque　as measured with indicators chosen a priori on the basis of clinical-physiological knowledge. Radiation was linearly associated with calcification (standardized coefficient per Gy 0.15, 95 % confidence interval: CI [0.070, 0.23]) and plaque (0.11, 95 % CI [0.029, 0.20]), small associations that were comparable to about 2 years of aging per Gy of radiation exposure, but not with arterial stiffness (0.036, 95 % CI [− 0.025, 0.095]). The model fitted better and had narrower confidence intervals than separate ordinary regression models explaining individual indicators independently. The associations were less evident when the dose range was restricted to a maximum of 2 or 1 Gy. By combining individual clinical-physiological indicators that are correlated because of common, underlying atherosclerotic pathologies, we found a small, but significant association of radiation with atherosclerosis.

**Supplementary Information:**

The online version contains supplementary material available at 10.1007/s10654-021-00731-x.

## Introduction

The potential effects of low-dose radiation on circulatory diseases are a global public health concern given the widespread use of diagnostic and interventional radiology. Epidemiological studies of persons exposed to radiation from radiotherapy [1, 2] have reported that radiation doses to the heart or mediastinum in excess of 2–4 Gy are associated with an elevated risk of cardiovascular disease (CVD). In addition, studies of the atomic bomb survivors [3, 4] have reported an association at lower doses (0.5 Gy and higher). There may also be evidence, although inconsistent, of increased mortality from CVD after exposure to even lower doses [5]. In contrast to higher doses (> 5Gy), the mechanisms of CVD related to such low-dose radiation are unclear [6]. Because CVD, such as coronary artery disease, develops on a background of atherosclerosis, systemic atherosclerosis subsequent to radiation-related tissue effects [7] is a possible mechanism. This potential mechanism, however, has not been investigated fully.

Atherosclerosis is a composite of pathological processes, which can be classified into three distinct, albeit interdependent, pathologies: (1) arterial stiffness, (2) calcification, and (3) plaque [8, 9]. Several subclinical measures of atherosclerosis have been reported to show independent predictive value for incident CVD events, including measures of plaque―carotid intima-media thickness (IMT) [10, 11], ankle-brachial index (ABI) [12–14], and upstroke time (UT) [15, 16], measures of calcification―X-ray examination of thoracic [17, 18] or abdominal [19] aorta, and measures of arterial stiffness―brachial-ankle pulse wave velocity (baPWV) [20, 21], augmentation index (AI) [22], and central systolic blood pressure (cSBP) [23]. Although any of these measures can be used as an indicator of atherosclerosis, each represents a partial aspect of their respective pathology. To assess overall atherosclerosis, therefore, multiple measures should be used. In addition, the data should be analyzed in a way that accounts for their correlations [24, 25], which come from measuring common or interdependent pathologies. In evaluating a given subclinical atherosclerotic measure, however, many studies have not accounted for the correlations with other measures, leaving the independence of the reported findings in doubt, and leaving the information of the correlations untapped.

Our aim was to examine the association between radiation dose and atherosclerosis using structural equation modeling to leverage the correlations of a comprehensive set of subclinical atherosclerotic measures including carotid IMT, ABI, UT, aortic calcification, baPWV, AI, and cSBP.

## Methods

This study is the first clinical survey of the atomic bomb survivors in Hiroshima and Nagasaki, Japan to make use of a comprehensive set of noninvasive measures including IMT, ABI, UT, baPWV, AI, cSBP, and aortic calcification evaluated from chest and lumbar X-ray examinations. The study was conducted as part of the Adult Health Study (AHS) of the Radiation Effects Research Foundation (RERF) between 2010 and 2014.

### Participants

In 1958, RERF established the AHS cohort in Hiroshima and Nagasaki, composed of 14,996 atomic bombing survivors and 5000 subjects who were not in the cities at the time of the bombings. The follow-up of the latter group was terminated in 1977; at the same time the cohort was expanded by adding 1185 newly identified subjects who were exposed to ≥ 1Gy radiation along with 1251 city-, age-, and sex-matched subjects exposed to < 1Gy radiation. In 1978, a total of 1021 survivors who were exposed in utero were added. In 2008, the cohort was expanded again by adding 1961 survivors exposed at less than 10 years of age; their estimated doses were relatively lower (mean 0.10 Gy). Health examinations have been performed biennially since 1958.

From 2010 to 2014, a total of 4782 cohort members participated in the AHS health examination. Out of them, participants without known hemodialysis were invited to participate in this study as part of their AHS examination as long as the daily capacity for atherosclerotic measurements permitted (n = 4123). After 247 refusers and 321 exposed in utero were excluded, 3555 participants (86.2 %) remained. Due to missing radiation dose estimates (n = 273) or missing smoking information (n = 8), there were 3274 participants available for analyses. Participants who had a history of vascular surgery (5 carotid artery, 16 peripheral artery, and 21 aorta) were included, but only the unaffected measurements were used for analysis depending on the surgical sites: carotid surgery could have effects on carotid IMT; peripheral artery surgery on ABI, UT, baPWV, AI, and cSBP; aortic surgery on ABI, UT, baPWV, AI, cSBP, and aortic calcification.

### Atherosclerotic indicators

Participants who agreed underwent the following measurements. In some participants, measurements were partial due to modality-wise refusal or to techinical difficulty (for example, difficulty in maintaining appropriate posture.)

#### ABI, UT, baPWV

A randomly assigned technician measured these indicators with a VP-2000 (Omron Health Care Co.; Kyoto, Japan), an automated oscillometric device that simultaneously records pulse waves at four limbs. ABI is the ratio of ankle to brachial systolic blood pressure, with the latter defined as the higher of that on either side. UT is time (msec) from the onset to the peak of the arterial pulse wave, and baPWV (m/sec) is assessed by dividing the travelled distance by the travel time between brachia and ankles. Because the calculations of baPWV and UT require precise identification of rise time, ABI measures only were obtained in some participants with questionable rise time (N = 26). In addition to prior vascular surgery, some measured values were considered invalid because of possible inaccurate measurement [26, 27]: ABI higher than 1.4 (N = 4 left side, 6 on right side) and baPWV if ABI on the same side was < 0.9 (N = 39 on the left side, 31 on the right side). Although ABI, UT, and baPWV values could be affected by heart rate, we did not adjust them for heart rate (such as dividing by cardiac cycle [16]) because such procedure could introduce artifactual correlation among the indicators.

#### AI and cSBP

AI and cSBP were measured in a quiet room with participants in sitting position after 10 minutes of rest. Measurements were made by trained technicians using an HEM-9000AI (Omron Health Care Co.), an automated oscillometric and tonometric device that simultaneously records blood pressure at the right brachial artery and a pulse wave at the left radial artery. From these records and additional anthropometric inputs, the device calculates AI and cSBP through a proprietary algorithm. The method has been validated by comparison with results of more invasive measures [28, 29]. In 46 participants, only AI was obtained because simultaneous barometry was unstable during measurement.

#### Calcification

Calcification was evaluated in both the thoracic and abdominal aorta by using chest and lumbar spine X-ray films, respectively. Calcification in the thoracic aorta was evaluated in the aortic arch, defined by the upper edge of the radiolucent image in the area from the trachea through the left main bronchus. The extent of calcification on a postero-anterior chest X-ray film was divided into four grades according to previously published criteria [18]: 0, no visible calcification; 1, small spots of calcification or a single thin area of calcification; 2, one or more areas of thick calcification; and 3, circular calcification.

Calcification in the abdominal aorta was evaluated in eight regions (the anterior and posterior walls at the level of L1–L4) on lateral lumbar spine X-ray films according to previously published criteria [30]. The extent of calcification was graded into four (0–3) grades in each of the eight regions, as follows: 0, no calcific deposits; 1, small scattered calcific deposits filling less than one-third of the longitudinal wall of the aorta; 2, one-third or more, but less than two-thirds of the longitudinal wall calcified; and 3, two-thirds or more of the longitudinal wall calcified. The eight individual grades were summed to create a total abdominal aorta calcification score.

#### Carotid artery IMT

Carotid artery IMTs (mm) in left and right sides were assessed by high-resolution B-mode ultrasound devices with 12 MHz linear probes: a LOGIQ S6 (GE Healthcare; Chicago, Illinois, United States) in Hiroshima, and a XarioXG (Toshiba; Tokyo, Japan) in Nagasaki. To obtain uniform images, depth was fixed at 4 cm, and dynamic range adjusted to 57 (LOGIQ S6) or 65 (XarioXG). The images obtained by the four fixed sonographers reveal (1) longitudinal lateral views of the far wall in the common carotid arteries (CCA) from 20 mm proximal to the tip of the flow divider, and (2) anterior, lateral and posterior views in the area ranging from the bifurcation to internal carotid arteries (ICA) beginning at the tip of the flow divider and extending 20 mm toward the ICA. All scans were performed according to the same scan protocol. Wall thickness was analyzed in each area using semi-automated software (Intimascope), and the maximal of all meaurements were used for each IMT.

### Covariates

Participants’ sex, city, and proximal-distal location at the time of bombing were obtained from the AHS cohort source data. Based on information periodically collected since 1963 through interviews and mail surveys, we categorized smoking status into three categories: “current” defined as those who were smoking at the time of examination (2010–2014), “past” as those who reported any smoking habit between 1963 and 2010 but not in 2010–2014, and “never” if otherwise.

### Radiation dose

The estimated radiation dose received by each participant was based on the latest atomic-bomb dosimetry system with recently improved inputs that provide for more accurate location and shielding by terrain at the time of the bombing (DS02R1) [31, 32]. Skin dose (shielded kerma, or whole-body, dose) was used in units of weighted Gy, with the dose to an individual being the sum of gamma ray dose plus ten times the smaller neutron dose [33]. Skin dose was selected a priori assuming that total body irradiation may affect the entire vascular system, such that it is difficult to identify another single organ dose that is appropriate. We used adjusted dose estimates that are intended to overcome regression bias that can arise from random uncertainties in exposure estimation [34].

### Statistical analyses

The data were analyzed by fitting a joint measurement and linear structural model relating the 14 measured indicators to the three underlying pathologies (arterial stiffness, calcification, and plaque), which were represented in the statistical model as latent factors, with covariates as causes of the factors—a multiple indicators, multiple causes (MIMIC) model [35–37].

**Fig. 1:**
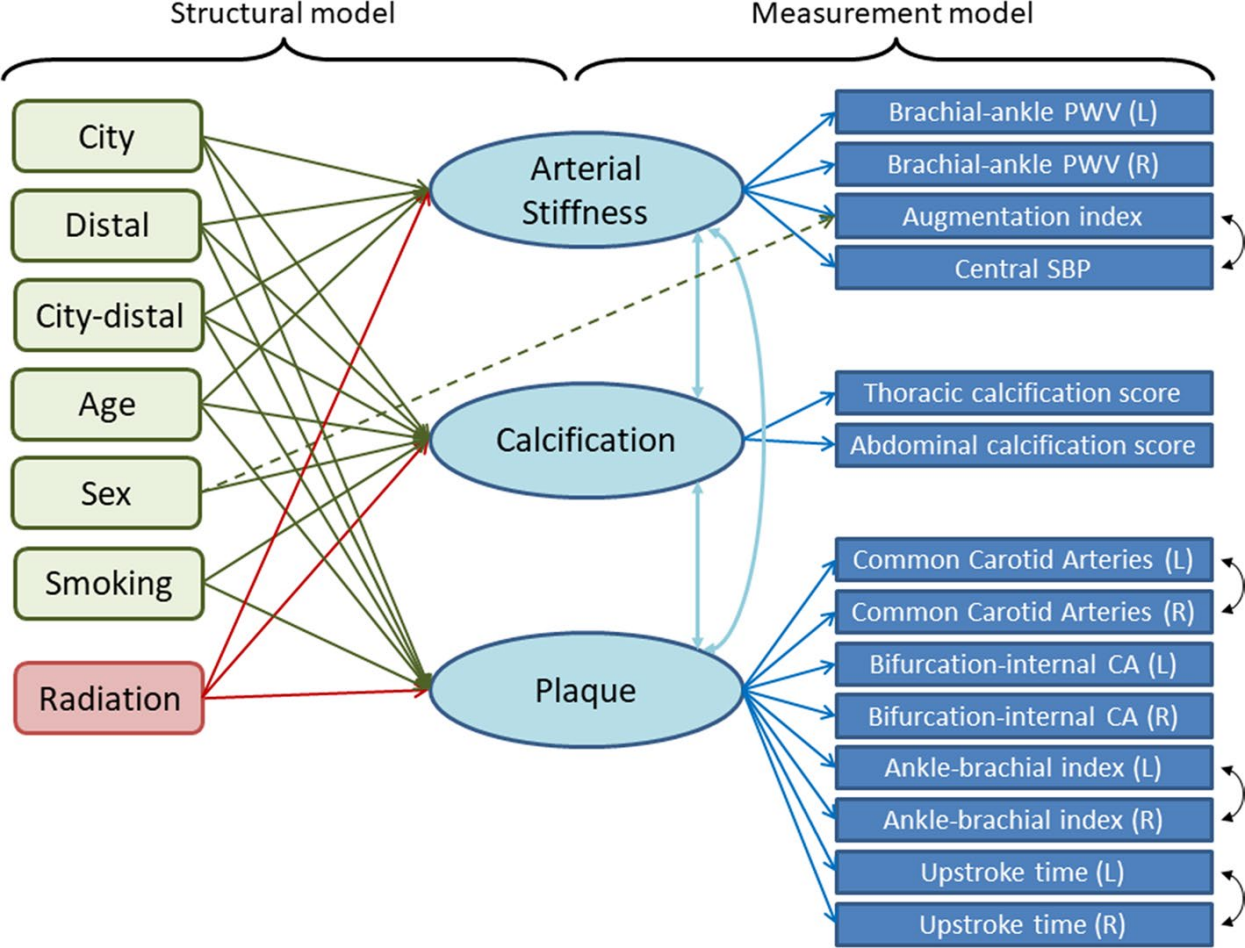
Diagram of final MIMIC model. Variables on the left are explanatory covariates that, together with the latent factors, make up the structural model. Variables on the right are measured clinical-physiological indicators of the latent atherosclerosis factors (the three variables contained in ovals in the center); the loadings of the indicators on the latent factors constitute the measurement model. The solid line paths (directed arrows) from covariates to latent factors represent linear effects that were supported by the data with concomitant adjustment for age, city, location at the time of exposure. The effect of age on calcification included a quadratic term as well as a linear term. The dashed line path represents a direct effect from a covariate to an indicator. Bi-directed paths (two-headed arrows) represent pairwise correlations included in the model

First, a measurement model relating the measured indicators to the underlying atherosclerotic pathologies (latent factors) was determined a priori on a physiological basis (Fig. 1, right half), and fit by confirmatory factor analysis. Although we assumed no causal paths among the latent factors, we introduced correlations among them to capture interdependence among the atherosclerotic pathologies including possible direct causation, such as increased stiffness by calcification. We also considered correlations among indicator residuals (correlations remaining after accounting for the shared pathologies) if such terms were indicated by large values of model modification indices and their addition improved model fit as judged by root mean square error of approximation (RMSEA) and comparative fit index (CFI) [37]; such correlations were retained only if their addition made clinical sense (e.g., shared measuring procedure). The variance of each latent factor was fixed at 1.0 to make the model parameters identifiable.

Second, a structural model was built to evaluate the association of radiation dose with each of the three latent factors, and to adjust for potential confounding (Fig. 1, left half). Along with age and sex, we included a priori as covariates the following variables that might act as surrogates of unmeasured confounding factors: city of residence (Hiroshima or Nagasaki), proximal-distal location (≤ or > 3 km from the hypocenter), and interaction between city and proximal-distal location at the time of the bombing. The interaction was included to allow for potential confounding between radiation dose and urban-rural location [38, 39], as the hypocenter was over the urban center in Hiroshima but more rural in Nagasaki. We also considered smoking behavior at the time of examination (current or past smoker, with never smoker as the reference category) as a proxy for potential confounding due to the possible association between pre-exposure socio-economic status (SES) and location, because smoking could mediate SES effects on CVD [40]. We expected that including smoking would not introduce substantial post-exposure bias because it is not caused by irradiation. However, to strive for parsimony, we retained smoking only if its coefficient was judged to be important, clinically or statistically, for any latent factor. Because the AHS expansion cohort, which consists of younger participants with lower radiation doses, could induce confounding of radiation effects by age, we carefully adjusted for age by including the square of age if it improved the fit of the model. Direct effects of covariates on indicators were included in the model if the effects were considered clinically plausible and if their addition was supported by the data as evidenced by substantial improvement in model fit.

We did not adjust for other known atherosclerosis risk factors, such as hypertension and cholesterol, to avoid bias caused by conditioning on mediators or colliders [41, 42]. While those risk factors might mediate some of the potential confounding through SES [40], they might also mediate radiation effects [43–45].　Thus they are both potential mediators and colliders. An attempt to adjust for potential confounding by including risk factors, therefore, runs the risk of canceling out indirect radiation effects mediated through them as well as bringing in additional confounding between radiation and SES (collider bias) [41, 42].

Interpretation of standardized coefficients in structural models is difficult because latent factors are unobserved and therefore have no meaningful scale. To help interpret radiation effects on atherosclerosis clinically, we estimated the effects of radiation on the unstandardized actual scale of the subclinical atherosclerotic indicators by taking the product of the corresponding structural and measurement parameters. With the assumption that there are no direct effects of radiation on the indicators, the indirect effects calculated in this way should represent the total effects estimated by the reduced form of the MIMIC model [46, 47].

For the sake of comparison, we also estimated total effects of radiation on the indicators by fitting ordinary regression analyses of the indicators on radiation (adjusted for the same variables as in the MIMIC model). To obtain fit indices comparable to those of MIMIC model, ordinary regression analyses were conducted collectively by assuming an uncorrelated multivariate normal distribution of all indicators, analogous to what would be obtained if separate regression analyses were conducted for each individual indicator.

To assess the validity of our model assumption that radiation effects on indicators pass through the specified latent factor, we also modified the full model to allow direct radiation effects on each individual indicator, in addition to mediation via the latent factors. To assess uncertainty as to potential unmeasured confounding, we calculated E-values [48] using the evalues. OLS( ) function in the R package EValue [49]. To assess potential selection bias pertaining to the latest expansion of the cohort in 2008, whose participation might have depended on different conditions from earlier sub-cohorts, we did an additional analysis excluding the latest expansion sub-cohort.

All continuous indicators were used without transformation in the analysis to allow for direct interpretation of estimated parameters except for re-scaling of some indicators to facilitate model convergence: ABI was multiplied by 10, and left and right baPWV were divided by 100. However, to assess the effects of departures from normality, we performed a supplementary analysis using natural log transformations of baPWV, IMT, and UT indicators. Although discrete, calcification scores were treated as continuous variables because no normalizing transformation was available. However, we assessed the effects of non-normality by applying proportional odds models to the calcification scores treated as ordinal categorical variables. Analyses were conducted with Mplus software [50] according to standard procedures [51, 52]. All models were fit by full information maximum likelihood assuming multivariate normality of the clinical indicators, which accommodates missing values under an assumption of missingness at random; hence, no participants with missing data were excluded except for the persons with unknown radiation dose or unknown smoking status. Confidence intervals are 95% intervals calculated by bootstrapping with 2,000 replications.

## Results

Among the 3274 eligible participants who were included in the analysis, mean DS02R1 skin dose was 0.30 Gy (SD 0.65, minimum 0.0 [381 participants], maximum 4.84). Participant characteristics across dose categories are summarized in Table 1. Among the categorical covariates, participants who were female, from Hiroshima, and proximally located at the time of the bombing were each in the majority. The differences between age at examination and age at radiation exposure across the dose strata reflects the expansion group added in 2008, which comprises 1703 people who are younger and have lower doses of radiation (mean age 70.8 years, mean dose 0.096 Gy) than the 1571 primary cohort (mean age 78.3 years, mean dose 0.52 Gy) (details provided elsewhere [53].)

**Table 1:** Analysis variable descriptive statistics by radiation dose categories and overall

Variable		Units	Number missing (out of 3274^a^)	Radiation dose category										Overall^a^ Mean: 0.301 Gy	
				0 to < 0.005 Gy Mean: 0.001 Gy		0.005 to < 0.5 Gy Mean: 0.116 Gy		0.5 to < 1 Gy Mean: 0.720 Gy		1 to < 2 Gy Mean: 1.36 Gy		2 + Gy Mean: 3.09 Gy			
				(n = 1163)		(n = 1529)		(n = 251)		(n = 229)		(n = 102)		(n = 3274)	
				Mean	SD	Mean	SD	Mean	SD	Mean	SD	Mean	SD	Mean	SD
*Continuous variables*^b^															
Age at examination		Years	0	75.3	6.3	72.6	5.3	76.9	7.0	77.9	6.5	76.6	7.0	74.4	6.2
Age at radiation exposure		Years	0	8.4	6.1	5.8	5.1	10.1	6.8	11.0	6.5	9.6	6.8	7.5	6.0
baPWV (L)		m/s^c^	370	18.7	3.7	17.9	3.7	19.4	4.2	19.1	4.3	19.5	4.2	18.4	3.8
baPWV (R)			365	18.7	3.9	17.8	3.6	19.2	4.1	19.1	5.2	19.3	4.1	18.3	3.9
Augmentation index			191	87.8	11.3	86.8	11.0	88.9	12.9	88.2	11.8	89.4	11.6	87.5	11.4
Central SBP		mmHg	236	140.9	19.8	140.2	19.9	141.4	21.2	139.0	19.3	144.8	24.6	140.6	20.1
Thoracic aorta calcification		grade	109	1.16	0.83	1.02	0.77	1.24	0.87	1.29	0.79	1.43	0.93	1.12	0.81
Abdominal aorta calcification			76	4.79	5.02	4.17	4.70	5.63	5.49	5.96	5.75	6.77	5.90	4.71	5.04
IMT in CCA (L)		mm	53	1.12	0.47	1.09	0.42	1.11	0.31	1.16	0.45	1.16	0.35	1.11	0.43
IMT in CCA (R)			46	1.08	0.38	1.06	0.33	1.08	0.33	1.12	0.40	1.21	0.49	1.08	0.36
IMT in ICA (L)		mm	160	1.71	0.90	1.69	0.86	1.78	0.96	1.82	1.00	1.79	0.95	1.71	0.90
IMT in ICA (R)			109	1.75	0.97	1.72	0.92	1.84	1.07	1.92	1.00	2.10	1.16	1.77	0.97
ABI (L)			311	1.13	0.081	1.13	0.080	1.13	0.077	1.12	0.080	1.12	0.095	1.13	0.080
ABI (R)			315	1.15	0.086	1.14	0.083	1.14	0.091	1.14	0.082	1.14	0.076	1.14	0.084
UT (L)		ms	331	143.9	24.3	144.6	24.5	147.3	26.8	148.3	28.3	152.0	31.1	145.0	25.1
UT (R)			334	144.3	24.0	144.4	24.4	146.8	24.2	148.3	27.7	150.7	28.9	144.9	24.7
*Categorical variables*^b^				n	%	n	%	n	%	n	%	n	%	n	%
Sex	Female			698	60.0	858	56.1	163	64.9	133	58.1	58	56.9	1910	58.3
	Male			465	40.0	671	43.9	88	35.1	96	41.9	44	43.1	1364	41.7
City	Hiroshima			575	49.4	1148	75.1	140	55.8	120	52.4	71	69.6	2,054	62.7
	Nagasaki			588	50.6	381	24.9	111	44.2	109	47.6	31	30.4	1220	37.3
Location ATB	Proximal (≤ 3 km)			269	23.1	1529	100.0	251	100.0	229	100.0	102	100.0	2380	72.7
	Distal (> 3 km)			894	76.9	0	0.0	0	0.0	0	0.0	0	0.0	894	27.3
Smoking status	Never			687	59.1	872	57.0	157	62.5	130	56.8	56	54.9	1902	58.1
	Current			97	8.3	149	9.8	20	8.0	19	8.3	2	2.0	287	8.8
	Past			379	32.6	508	33.2	74	29.5	80	34.9	44	43.1	1085	33.1

ABI, ankle-brachial index; ATB, at the time of the bombing; baPWV, brachial-ankle pulse wave velocity; CCA, common carotid arteries; ICA, internal carotid arteries; IMT, intima-media thickness of the carotid artery wall; NA, not applicable; SD, standard deviation; UT, upstroke time

^a^Numbers of missing values are among participants with known radiation doses and known smoking status, the sample used in the analyses. All reasons for missing values, including not measured and invalid measurement, are combined. Overall variable summaries (not stratified by dose group, final two columns) are for this same sample of participants

^b^Continuous variables are summarized by mean and standard deviation (SD). Categorical variables are summarized by number of participants and proportion (%). No categorical variables other than smoking had unknown values

^c^Original baPWV measurements were made in cm/sec

All of the continuous indicator variables had approximately normal distributions except for the IMT variables, which had only slight skewness. As for the discrete indicators (thoracic and abdominal calcification scores), there is no transformation that can produce approximate normality given that their modes are zero, so they were left untransformed. Histograms of the distributions of the indicators are provided in Supplemental Figure S1. Because the fit of the MIMIC model is based on the covariance structure among the measured indicators, correlations among the indicators are shown in Supplemental Figure S2.

The final fitted MIMIC model was composed of a measurement model and a structural model (shown on the right and left halves of Fig. 1, respectively). Standardized parameters from the MIMIC model are shown in Table 2.

**Table 2:** Standardized parameter estimates from the fit of the MIMIC model

Sub-model	Latent atherosclerotic factor	Parameter	Estimated value	95 % CI
Measurement model	Arterial stiffness	baPWV (left)	0.98	0.97, 1.00
(right half of Fig. 1)		baPWV (right)	0.97	0.96, 0.98
		AI	0.11	0.07, 0.14
		cSBP	0.42	0.37, 0.46
	Calcification	Thoracic aorta	0.48	0.44, 0.51
		Abdominal aorta	0.84	0.80, 0.89
	Plaque	CCA IMT (left)	0.39	0.34, 0.44
		CCA IMT (right)	0.43	0.38, 0.47
		ICA IMT (left)	0.64	0.60, 0.68
		ICA IMT (right)	0.67	0.63, 0.71
		ABI (left)	−0.21	−0.27, −0.15
		ABI (right)	−0.24	−0.29, −0.18
		UT (left)	0.40	0.35, 0.46
		UT (right)	0.42	0.37, 0.47
	Arterial stiffness with Calcification	Correlation coefficient	0.24	0.20, 0.29
	Arterial Stiffness with Plaque		0.08	0.03, 0.13
	Calcification with Plaque		0.65	0.59, 0.71
	Other components	Direct effect of sex on AI	0.78	0.72, 0.83
		Indicator correlations		
		cSBP and AI	0.47	0.43, 0.50
		CCA IMT (left and right)	0.30	0.23, 0.37
		ABI (left and right)	0.66	0.63, 0.70
		UT (left and right)	0.80	0.77, 0.83
Structural model	Arterial Stiffness (*R*^2^ = 0.21)	Radiation dose (per Gy)	0.036	−0.025, 0.095
(left half of Fig. 1)		1 year of age (linear)	0.074	0.069, 0.079
		City ^a^	0.017	−0.067, 0.11
		Distal indicator	−0.094	−0.18, −0.008
		City-distal interaction	0.17	0.024, 0.31
	Calcification (*R*^2^ = 0.27)	Radiation dose (per Gy)	0.15	0.070, 0.23
		1 year of age (linear)	0.062	0.054, 0.069
		(quadratic)	0.001	0.001, 0.002
		Female	0.19	0.091, 0.29
		City ^a^	−0.18	−0.27, −0.090
		Distal indicator	0.11	−0.007, 0.23
		City-distal interaction	−0.085	−0.26, 0.090
		Smoking (current)	0.47	0.36, 0.58
		(past)	0.54	0.38, 0.68
	Plaque (*R*^2^ = 0.28)	Radiation dose (per Gy)	0.11	0.029, 0.20
		1 year of age (linear)	0.057	0.050, 0.064
		City^a^	−0.50	−0.58, −0.41
		Distal indicator	0.083	−0.048, 0.22
		City-distal interaction	−0.003	−0.18, 0.17
		Smoking (current)	0.48	0.39, 0.57
		(Past)	0.63	0.48, 0.79

Each parameter corresponds to each path or correlation (unidirectional or bidirectional arrow) in Fig. 1

Standardized to SDs of latent factors and indicators, but not covariates

ABI, ankle-brachial index; AI, augmentation index; cSBP, central systolic blood pressure; CCA, common carotid arteries; ICA, bifurcation to internal carotid arteries; IMT, intima-media thickness; baPWV, brachial-ankle pulse wave velocity; UT, upstroke time

^a^City effects are effects in Nagasaki (with Hiroshima as the reference group)

The overall fit of the measurement model (before including covariates) was acceptable, with CFI = 0.943 and RMSEA = 0.067. In the measurement model (Table 2, upper half and Fig. 1, right half), baPWV was an excellent indicator for arterial stiffness, as indicated by a large standardized coefficient. Abdominal aorta calcification performed better than thoracic as an indicator of calcification, as did IMT and UT as indicators of plaque compared to ABI. Despite shared latent factors, the resulting model included correlations between AI and cSBP, between left and right CCA IMT, between left and right ABI, and between left and right UT (Fig. 1; Table 2). These correlations presumably reflect shared measurement errors or anatomical resemblance. Although similar inter-side correlation was expected for baPWV, introducing it degraded the model presumably because of their high factor loadings.

In the structural model (Table 2, lower half and Fig. 1, left half), which predicts latent factors (underlying atherosclerotic pathologies) by radiation and other covariates, radiation dose was significantly associated with calcification and plaque, but not with arterial stiffness. As for the other covariates, arterial stiffness was significantly associated with age and location; calcification with age, age squared, sex, city, and smoking; plaque with age, city, and smoking. A direct effect of sex on AI, reported previously [54], was confirmed in our cohort; it was the only direct effect of a covariate on an indicator suggested by the data as expected from the absence of other such reports. All of the latent factor inter-correlations were statistically significant, with the most notable correlation observed between calcification and plaque (0.65), followed by that between arterial stiffness and calcification (0.24), and arterial stiffness and plaque (0.080). Although the addition of covariates led to some degradation in model fit indices (as has been noted by others [36]), it caused no substantial changes in factor loadings.

The estimated linear associations between 1 Gy radiation and the two latent factors calcification and plaque were comparable to about 2 years higher age (Table 2). The standardized coefficient for the regression of calcification on radiation dose (0.15 per Gy [95 % CI, 0.070, 0.23]) was about 2.4 times that of 1 year higher age (0.062); the standardized coefficient for the regression of plaque on radiation dose (0.11 per Gy [95 % CI, 0.029, 0.20]) was about two times that of 1 year higher age (0.057). Quadratic terms of radiation were not significant for any of the three latent atherosclerotic factors (Supplemental Table S1). When the data were restricted to participants with doses of 2.0 Gy or less (N = 3172), the association became less evident although the estimated radiation effects were not greatly different: 0.11 (95 % CI [−0.014, 0.23]) for calcification and 0.11 (95 % CI [0.001, 0.23]) for plaque. When the data were restricted to participants with doses of 1.0 Gy or less (N = 2943), corresponding values were 0.17 (95 % CI [–0.039, 0.38]) and 0.092 (95 % CI [−0.11, 0.30]), respectively (Supplemental Figure S3). The E-values for the association of radiation dose with plaque and calcification were 1.51 (lower limit 1.21) and 1.61 (lower limit 1.36) respectively.

**Fig. 2:**
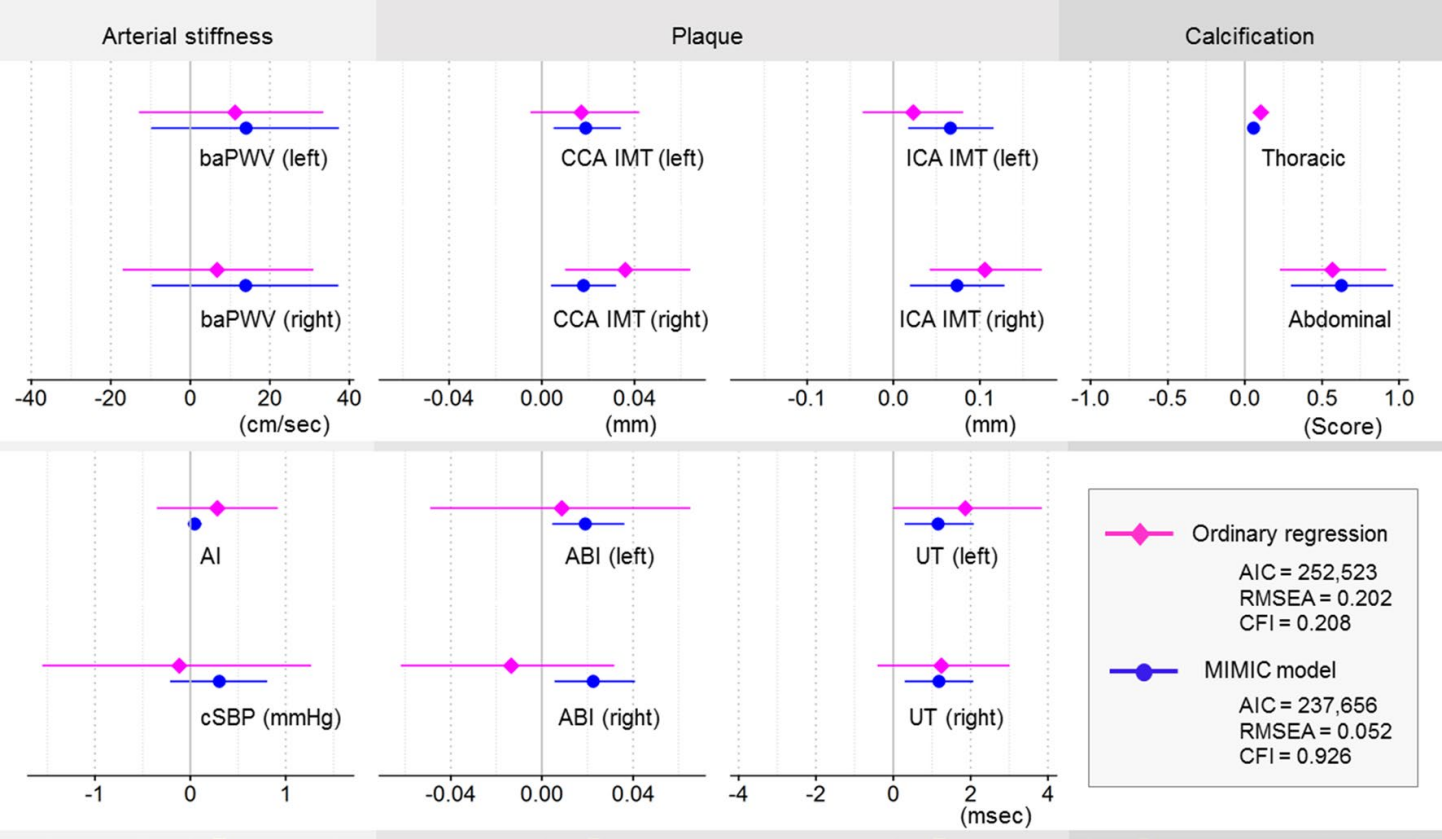
Estimated effects of radiation (per Gy) on atherosclerosis indicators: comparing the MIMIC model with ordinary regression. Changes (decrease in ABI and increase in the others) in indicators per Gy of radiation dose are shown as point estimates with 95% confidence intervals (CI). The MIMIC model performs better in terms of model fit (lower AIC and RMSEA, higher CFI), consistency (greater agreement and stability among common measurements), and presumably efficiency (narrower CIs). Estimates were adjusted for the covariates specific to each latent atherosclerotic factor in the MIMIC model (Fig. 1): for the arterial stiffness indicators, age, city, distal, and city-distal interaction (and a direct effect of sex on AI); for the calcification indicators, age, age-squared, sex, city, distal, city-distal interaction, and smoking; for the plaque indicators, age, city, distal, city-distal interaction, and smoking. baPWV, brachial-ankle pulse wave velocity; AI, augmentation index; cSBP, central systolic blood pressure; IMT, intima-media thickness; CCA, common carotid arteries; ICA, bifurcation to internal carotid arteries; ABI, ankle-brachial index; UT, upstroke time; AIC, Akaike information criterion; RMSEA, root mean square error of approximation; CFI, comparative fit index

To facilitate interpretation of the MIMIC model results, we calculated unstandardized estimated effects of radiation on the indicators and compared them with ordinary regression analyses (Fig. 2) and with a multivariate regression model (Supplemental Figure S4). The estimated effects from the MIMIC model were mostly comparable to those estimated by ordinary regression, but provided more stable estimates, as shown by greater consistency in effects observed for the same indicator on each side of the body. The MIMIC model fitted better, as shown by higher CFI and lower RMSEA and AIC, and had narrower confidence intervals.

To validate the assumption that there are no remaining direct effects of radiation on each indicator beyond the indirect relationship through the latent variables, we tested whether any significant direct effects of radiation were observed for each of the 14 indicators individually in the MIMIC model. After Bonferroni correction to account for multiple tests (i.e., a modified *p*-value significance level of 0.003), only one clinical indicator (left ICA IMT) showed a significant direct radiation effect; in addition to a significant increase of 0.091 (95 % CI [0.039, 0.15]) per Gy of radiation dose indirectly mediated through the plaque latent factor, the IMT measurement of left ICA directly decreased by 0.073 (95 % CI [0.027, 0.12]). Because a unilateral radiation effect in only one indicator is biologically implausible, we believe that the statistical significance of this small direct effect was a chance finding and that assuming no direct effects was mostly supported by the data.

When the continuous indicators with apparent skewness (baPWV, IMT, and UT) were log-transformed, estimated radiation effects were nearly identical (effects on calcification, plaque, and arterial stiffness per Gy were 0.15 (95 % CI [0.071, 0.23]), 0.11 (95 % CI [0.032, 0.18]), and 0.034 (95 % CI [−0.026, 0.091]), respectively. When the members of the latest expansion sub-cohort (N = 1703) were excluded, there were no substantial changes in estimated radiation effects (supplemental Table S2). When calcification scores were treated as ordinal categorical variables, they were significantly associated with radiation exposure (Supplemental Table S3).

## Discussion

In this study, we modeled three known atherosclerotic pathologies as latent factors, which combined multiple correlated clinical-physiological indicators of atherosclerosis via the common underlying pathologies. This model, as long as reasonably specified, increased efficiency for assessing the radiation effect, wherewith we found significant associations of radiation with two of the three pathologies, calcification and plaque, reinforcing the evidence for radiation effects on atherosclerosis. On the other hand, we did not find a significant association with arterial stiffness.

This is the first clinical survey of the atomic bomb survivors to make use of a comprehensive collection of noninvasive measures. This study confirmed the significant association between radiation and aortic calcification found in a prior study on the atomic-bomb survivors [55]. In addition, this study demonstrated a statistically significant association with atherosclerotic plaque as indicated by the observed values of IMT, ABI, and UT. In the prior study [55], the association of radiation with common carotid artery IMT was comparable to slightly more than that of 1 year of age, but statistically non-significant. Increased power due to a larger sample and use of multiple indicators might have enabled this study to detect small associations, which were equivalent to about two years of age and to about one-fifth those of smoking.

Unfortunately, we are not able to directly compare the magnitude of the radiation effects to those of other traditional risk factors such as hypertension because we were not able to include most of them due to their potential roles as mediators. However, we might make the following indirect inference. First, in this study, the association of plaque with smoking was equivalent to 10 years of age. Second, in previous studies, the associations of carotid IMT with smoking and with other traditional risk factors were also equivalent to about 10 years of age [56, 57]. Consequently, the magnitude of the radiation effects could be comparable to about one-fifth those of traditional risk factors. Such an indirect comparison requires hesitation, however, because the coefficients obtained from different models may not be comparable [58]. In particular, the coefficient of age in this study might include some of the effects of traditional risk factors, which generally become prevalent with increasing age. In addition, it is important to remember that total radiation effects detected in this study might not be independent of traditional risk factors because some of the effects could have been mediated through traditional risk factors, such as hypertension [43, 44] and cholesterol [45].

Atherosclerosis is a complex process that involves different pathologies, which can be classified into plaque, calcification, and arterial stiffness [8, 9]. Although not fully elucidated, these pathologies seem to have distinct molecular mechanisms with some degree of co-regulation [8, 9, 59, 60]. Our study provides evidence for such structure as shown by the degree of correlation among the three latent factors. Furthermore, these pathologies were associated with radiation in different ways: the association was larger with plaque and calcification than with arterial stiffness. This difference suggests that radiation exerts greater effects on processes involving active proliferation and differentiation than on the disintegration of arterial wall elastic fibers.

Because those mechanisms, similar to aortic calcification, are implicated in the pathogenesis of aortic valvular disease [61], the association between radiation and calcification in the present study is concordant with previous studies in the atomic bomb survivors [3, 4] that have demonstrated increased mortality from valvular heart disease. On the other hand, the association with plaque in this study seems contradictory to the lack of observed association thus far between radiation and mortality from ischemic heart disease [3, 4]. This apparent paradox may be partially explained by the relatively small degree of association detected in this study (comparable to about two years of aging). It may also be partially explained by the lack of an observed association with arterial stiffness in the present study. Another potential explanation lies with the possible dual character of plaque calcification: it may stabilize or destabilize atheromatous plaques depending on the circumstances [62].

In this study, radiation was significantly associated with both indicators of calcification in ordinary regression analysis. Also, the associations with plaque indicators were positive in seven out of eight, among which two were statistically significant. Although this tendency suggests radiation effects on plaque, the inconsistent associations among the plaque indicators appears to contradict the putative biological mechanisms that radiation should affect the underlying mechanism, not its indicators directly. This inconsistency was resolved by the use of structural equation modeling. In addition, it produced narrower CIs. These differences presumably come from modeling the underlying interdependent pathologies. Such models can borrow information across multiple correlated indicators in a biologically plausible way [63] and accommodate missing indicators relatively well [64, 65], in contrast to ordinary regression, which incorrectly assumes independent radiation effects on indicators and uses only information on one indicator at a time. These improvements, although dependent on model specification, suggest the advantage of structural equation modeling over ordinary regression analysis when there are complex relationships such as common underlying mechanisms.

When interpreting the results of the structural equation model, however, a caveat should be noted. Although we carefully built the model on the basis of biological and clinical considerations, and checked the model assumptions by verifying no evidence of direct effects, there remains a possibility of misspecification that could have impacted the estimation. In particular, the MIMIC model without direct effects of covariates on indicators constrains the radiation effect to pass through the specified latent factor (plaque in the case of ABI, for example). On the other hand, ordinary regression does not impose such constraints; effects of radiation could pass through any, all, or no latent factors. Therefore, some of the differences between the MIMIC model and ordinary regression results (Fig. 2) could stem from our constraints regarding how the indicators depend on the latent factors. This might have been the case with some physiological indicators. For example, ABI (used as an indicator of plaque) increases when the arteries in lower extremities are heavily calcified [27], which suggests a potential effect on ABI through calcification, in addition to that through plaque. Although the presence of such cross-loadings was not supported by the lack of direct effects of radiation on indicators, it remains a possibility. On the other hand, a similar caveat would not apply to the imaging indicators (calcification scores and carotid IMT). This is because brightness in the X-ray film is solely determined by the content of calcium, which is not affected by plaque or stiffness. Similarly, IMT is a direct imagining method for detecting plaque.

In this study, the radiation associations were less clearly established when subjects were limited to those exposed to radiation doses under 1 or 2 Gy (Supplemental Figure S3). This lack of significance could simply be due to decreased sample size given that the magnitude of the effect sizes remained largely unchanged with marginal significance when dose ranges were limited; a linear dose-response may well hold in the low-dose range. Nevertheless, the widths of confidence intervals could also allow for a non-linear relationship. Although the quadratic model was not favored over the linear model (Supplemental Table S1), there might be some other form of non-linear dose response not well captured by a global quadratic term over the entire dose range. In any case, the associations are expected to be small in these low-dose ranges, probably comparable to 2 years of age or less. Such small associations, if any, are difficult to demonstrate unambiguously even with this presumably efficient statistical method. To clarify the potential effects of radiation at lower dose ranges, further studies are required.

Our modeling is analogous to the reasoning of clinicians who infer atherosclerosis in their patients by integrating multiple clinical indicators. Clinicians measure multiple indicators with a hope that it would assess overall atherosclerosis and prognosticate cardiovascular diseases better than measuring a single indicator. Unfortunately, there has been no reliable way of cardiovascular prognostication by combining multiple atherosclerosis indicators. If our model indeed captured much of the real pathophysiology, using latent variables as predictors rather than single indicators separately could improve prognostication, as was demonstrated in clinical prediction using linear models [63]. This is a potentially fruitful topic for future study.

Strengths of our study include the following. First is that it was conducted within a clinical cohort with high participation rate. Second is the large sample size. Third is the measurement of a large number of clinical indicators of atherosclerosis, some of which are not routinely available in clinical settings. Fourth is our use of a sophisticated model that combines correlated clinical indicators through their common dependence on underlying atherosclerotic pathologies. Such model can provide consistency and better statistical efficiency than would be achieved by separate analyses of individual indicators on the assumption that the model was reasonably specified, an assumption that we examined carefully.

Limitations of our study include uncertainties inherent in our cross-sectional study of this observational cohort. The first is possible selection bias from attrition after many decades since radiation exposure. This could underestimate radiation effects because of higher mortality among those with more severe atherosclerosis. The second is possible residual confounding by unmeasured confounders. One such factor could be pre-exposure SES, such as education, income, and employment status, which could be associated with radiation dose through location. To control for such potential confounding, we attempted to crudely adjust for this by including city, proximal/distal, their interaction, and smoking. The E-values [49] for the association of radiation with plaque and calcification were 1.51 (lower limit 1.21) and 1.61 (lower limit 1.36) respectively. This indicates a residual confounder with the relative risk of that magnitude for both radiation dose and atherosclerosis independent of measured variables could—but not necessarily would—explain away the observed association, although the relative risk for a latent factor could be less clearly interpretable compared with directly measurable factors. Apart from potential bias, it should be mentioned that the estimated radiation effects in this study may include “non-radiation” effects because atherosclerosis can be affected by compromised SES or psychological strain caused by devastation from the bombing. A third limitation is that we did not include other traditional health-related risk factors, such as hypertension, which were measured after exposure, although they could mediate part of the potential confounding by SES [40]. This is because they could also mediate radiation effects; if the analysis conditioned on them, it might not only underestimate radiation effects but also introduce collider bias [41]. In the future we hope to identify appropriate indicators that will allow us to add these outcomes to the structural model and test for mediation.

## Conclusion

We demonstrated the association between radiation dose and two out of three kinds of atherosclerotic pathologies, calcification and plaque (but not arterial stiffness), by applying structural equation modeling to a set of multiple correlated clinical indicators. The results of this cross-sectional study suggest a possible causative role of radiation on atherosclerosis, which should be confirmed by future longitudinal studies.

## Supplementary Information

Below is the link to the electronic supplementary material.

Supplementary Material 1

## Data Availability

The data will be made available by request to RERF after ethical and scientific institutional review although we cannot publicly share the data or indiscriminately provide them upon request to protect privacy of atomic-bomb survivors. Those interested can contact the corresponding author.
