# Supplementary material for: Radiation effects on atherosclerosis in atomic bomb survivors: a cross‐sectional study using structural equation modeling
Source: Eur J Epidemiol. 2021 Mar 19;36(4):401–14. doi: 10.1007/s10654-021-00731-x (PMC8076141; doi:10.1007/s10654-021-00731-x)
Supplement: Supplementary file 1 — Supplementary Material 1 [file 10654_2021_731_MOESM1_ESM.docx]

# **SUPPLEMENTAL MATERIAL**

**Supplemental Figure S1. Distributions of measured atherosclerosis indicators**

Distributions are shown for the analysis sample of 3,274 participants with known radiation dose and known smoking status.

***Indicators of arterial stiffness***

***Indicators of aortic calcification***

***Indicators of plaque***

Note: high outlying values of IMT that are not clearly visible on the histograms include: 6.21 and 7.42 for left CCA; 6.02 for right CCA; 6.30, 6.37, 6.76, and 7.01 for left ICA; and 6.93, 7.37, 7.46, and 8.70 for right ICA.

Note: low outlying values of ABI that are not clearly visible in the histograms include: 0.63, 0.70 (×2), and 0.72 for left ABI; 0.52, 0.59, and 0.64 (×2) for right ABI.

Note: high outlying values of UT that are not clearly visible in the histograms include: 272, 281 (×2), and 301 for left UT; 273, 283, 294, and 297 for right UT.


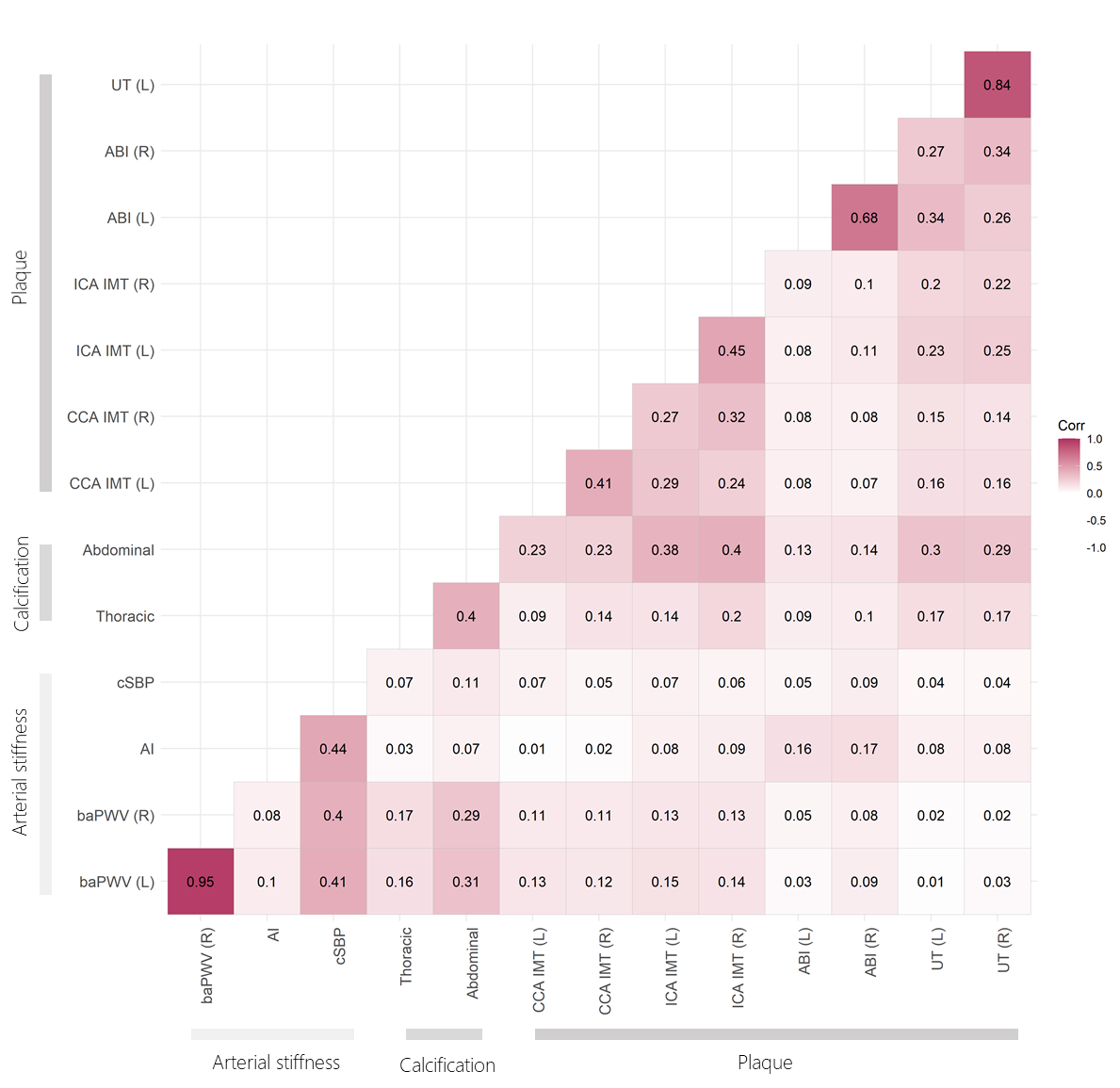


**Supplemental Figure S2. Correlations among clinical indicators**

The correlations are calculated on a pair-wise basis. The signs of ABIs are inverted for the sake of comparison.

Abbreviations: ABI, ankle-brachial index; AI, augmentation index; cSBP, central systolic blood pressure; CCA, common carotid arteries; ICA, bifurcation to internal carotid arteries; IMT, intima-media thickness; baPWV, brachial-ankle pulse wave velocity; UT, upstroke time.

**Supplemental Table S1. Comparison of linear and linear-quadratic models**

|  | Linear (95% CI) | Linear-quadratic (95% CI) |
| --- | --- | --- |
| Radiation effect (per Gy) |  |  |
| Arterial stiffness |  |  |
| Linear | 0.036 (−0.025, 0.095) | −0.059 (−0.209, 0.082) |
| Quadratic | NA | 0.032 (−0.011, 0.078) |
| Calcification |  |  |
| Linear | 0.148 (0.070, 0.228) | 0.017 (−0.151, 0.183) |
| Quadratic | NA | 0.043 (−0.014, 0.096) |
| Plaque |  |  |
| Linear | 0.114 (0.029, 0.201) | 0.040 (−0.132, 0.225) |
| Quadratic | NA | 0.024 (−0.042, 0.085) |
|  |  |  |
| Model fit indices |  |  |
| AIC | 237,656 | 237,656 |
| RMSEA | 0.052 (90% CI = 0.050, 0.054) | 0.050 (90% CI = 0.048, 0.053) |
| CFI | 0.926 | 0.925 |

**Supplemental Table S2. Selected standardized parameter estimates from the MIMIC model excluding the latest expanded sub-cohort. (N = 1,571)**

| **Latent atherosclerotic factor** | **Parameter** | **Estimate** | **95% CI** |
| --- | --- | --- | --- |
| Arterial Stiffness | baPWV (left) | 0.99 | 0.96, 1.01 |
|  | baPWV (right) | 0.96 | 0.94, 0.98 |
|  | AI | 0.13 | 0.08, 0.19 |
|  | cSBP | 0.43 | 0.36, 0.49 |
| Calcification | Thoracic aorta | 0.50 | 0.45, 0.55 |
|  | Abdominal aorta | 0.84 | 0.78, 0.91 |
| Plaque | CCA IMT (left) | 0.37 | 0.30, 0.45 |
|  | CCA IMT (right) | 0.42 | 0.35, 0.48 |
|  | ICA IMT (left) | 0.64 | 0.59, 0.69 |
|  | ICA IMT (right) | 0.68 | 0.63, 0.73 |
|  | ABI (left) | −0.28 | −0.36, −0.20 |
|  | ABI (right) | −0.30 | −0.38, −0.22 |
|  | UT (left) | 0.45 | 0.38, 0.52 |
|  | UT (right) | 0.47 | 0.40, 0.53 |
| Arterial Stiffness with Calcification  Arterial Stiffness with Plaque  Calcification with Plaque | Correlation coefficient | 0.17  0.03  0.67 | 0.11, 0.24  −0.05, 0.10  0.60, 0.75 |
|  |  |  |  |
| Arterial Stiffness (*R*^2^=0.21) | radiation dose (per Gy) | 0.023 | −0.045, 0.092 |
|  | 1 year of age (linear) | 0.068 | 0.062, 0.075 |
|  | city ^a^  distal indicator  city-distal interaction | −0.077  −0.17  0.30 | −0.22, 0.070  −0.30, −0.027  0.086 0.50 |
|  |  |  |  |
| Calcification (*R*^2^=0.22) | radiation dose (per Gy) | 0.13 | 0.041, 0.21 |
|  | 1 year of age (linear)  (quadratic) | 0.054  0.001 | 0.044, 0.065  0.000, 0.002 |
|  | female | 0.29 | 0.15, 0.44 |
|  | city ^a^  distal indicator  city-distal interaction | −0.22  0.072  −0.086 | −0.35, −0.076  −0.087, 0.23  −0.32, 0.14 |
|  | smoking (current)  (past) | 0.38  0.56 | 0.22, 0.53  0.34, 0.81 |
|  |  |  |  |
| Plaque (*R*^2^=0.23) | radiation dose (per Gy) | 0.090 | 0.002, 0.18 |
|  | 1 year of age (linear) | 0.048 | 0.040, 0.058 |
|  | city ^a^  distal indicator  city-distal interaction | −0.56  0.012  0.066 | −0.69, −0.43  −0.17, 0.19  −0.17, 0.30 |
|  | smoking (current)  (past) | 0.42  0.72 | 0.29, 0.55  0.48, 0.97 |

Standardized to SDs of latent factors and indicators, but not covariates.

^a^ City effects are effects in Nagasaki (with Hiroshima as the reference group).

Abbreviations: ABI, ankle-brachial index; AI, augmentation index; cSBP, central systolic blood pressure; CCA, common carotid arteries; ICA, bifurcation to internal carotid arteries; IMT, intima-media thickness; baPWV, brachial-ankle pulse wave velocity; UT, upstroke time.

**Supplemental Table S3.** **Ordinal logistic regression for calcification indicators**

| **Model Type** | **Factor** | **Variable** | **Estimate** | **95% CI** |
| --- | --- | --- | --- | --- |
| MIMIC model with categorical calcification indicators | Calcification | Thoracic aorta | 0.49 | 0.45, 0.53 |
|  |  | Abdominal aorta | 0.81 | 0.76, 0.86 |
|  | Arterial Stiffness with Calcification | Correlation  coefficient | 0.17 | 0.11, 0.24 |
|  | Calcification with Plaque |  | 0.67 | 0.60, 0.75 |
|  | Calcification (*R*^2^=0.25) | radiation dose (per Gy) | 0.16 | 0.081, 0.23 |
|  |  | 1 year of age (linear) | 0.063 | 0.055, 0.072 |
|  |  | (quadratic) | 0.001 | 0.000, 0.002 |
|  |  | female | 0.19 | 0.083, 0.30 |
|  |  | city ^b^ | −0.17 | −0.27, −0.071 |
|  |  | distal indicator | 0.15 | 0.021, 0.27 |
|  |  | city-distal interaction | −0.10 | −0.28, 0.079 |
|  |  | smoking (current) | 0.50 | 0.39, 0.62 |
|  |  | (past) | 0.57 | 0.42, 0.73 |
| **Model Type** | **Outcome** | **Variable** | **OR** | **95% CI** |
| Separate ordinal logistic regressions | Thoracic aorta | radiation dose (odds ratio at 1 Gy) | 1.27 | 1.12, 1.45 |
|  | Abdominal aorta |  | 1.20 | 1.06, 1.36 |

To assess the sensitivity of our results to non-normality of calcification scores, we ran an alternative MIMIC model treating the calcification indicators as ordinal categorical variables. Thoracic scores were treated as 4 graded categories as originally measured. Abdominal scores were coarsened into 7 categories (0: score 0, 1: score 1–4, 2: score 5–8, … , 6: score 21–24) to allow stable estimation. These standardized estimates and bootstrap confidence intervals are comparable to those shown in Table 2 and Table S2. Furthermore, to assess the direct association of radiation with the two calcification outcomes considered as ordinal categorical variables, we have included the results from independent ordinal logistic regressions which ignore the correlation of these outcomes (similar to the separate ordinary regressions comparison in Figure 2). Although not directly comparable due to differences in scale (multiplicative versus additive), there was consistency in our observation of significant radiation effects among MIMIC and ordinary regression models for continuous, log-transformed, and categorical outcomes. This consistency suggests insensitivity to non-normality.


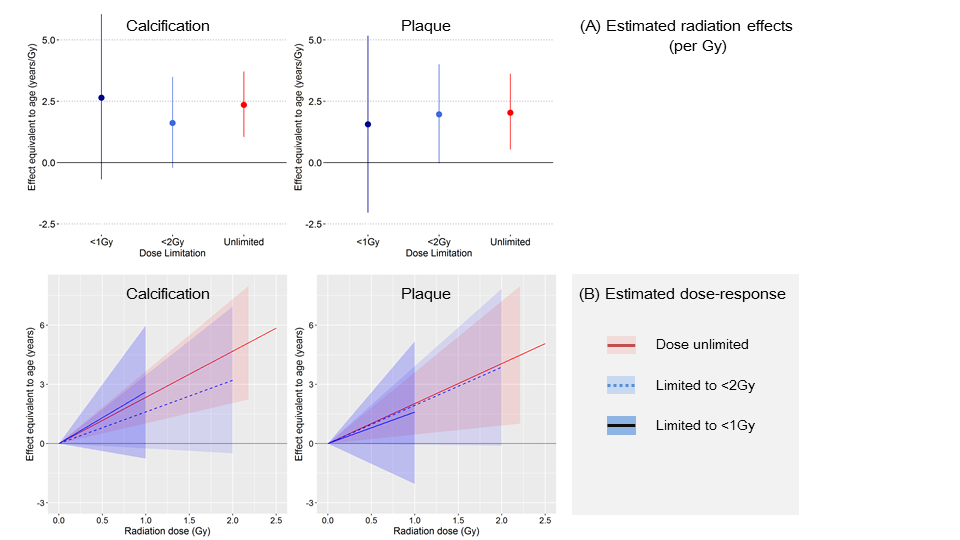


**Supplemental Figure S3. Comparison of linearly approximated radiation effects estimated by subjects with or without limitation to lower doses.**

(A) Estimated radiation effects per Gy (B) Estimated dose-response

Point estimates with 95% confidence intervals are shown.

For comparison, estimated radiation effects were “standardized” to age, calculated as the coefficient ratio to age and expressed as the effects equivalent to age (years). Estimates were obtained through Monte Carlo simulation using estimated mean and standard error of the coefficients.


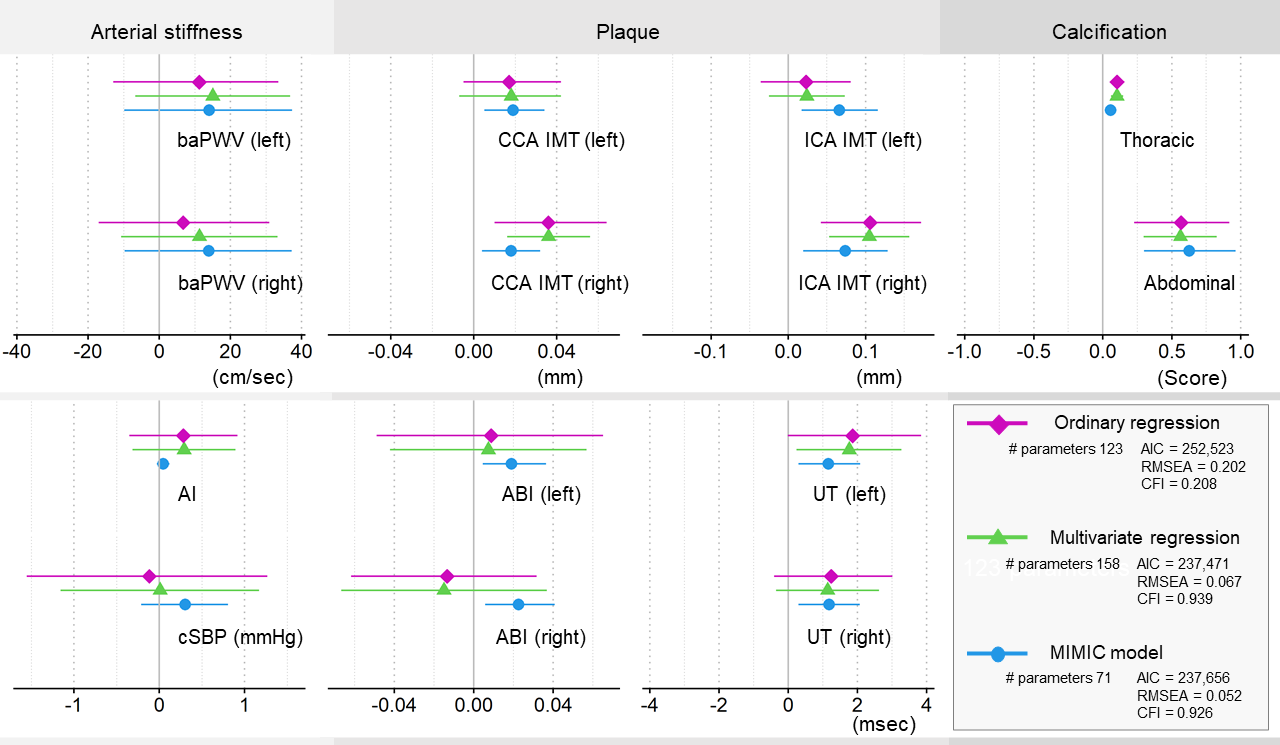


**Supplemental figure S4. Comparison with multivariate normal regression**

Although we primarily compared structural equation modeling (SEM) with a set of independent ordinary regression models on individual indicators—a common analysis in clinical studies, we also compared with another possible approach that could accommodate correlation among indicators—fitting a set of three multivariate normal regressions, one for each of the underlying pathologies (grouped multivariate model).

As shown in the figure, the grouped multivariate model generated estimates which were largely the same as the separate ordinary regressions. This similarity presumably comes from the shared assumptions: 1) independence among arterial stiffness, calcification, and plaque, and 2) independent effects of radiation on each of the indicators.

Although the model fit indices are improved, the grouped multivariate model narrowed CIs only slightly. This superficial improvement in fit indices presumably stems from inefficient over-parametrization, which wasted information in estimating nuisance parameters (separate covariances between pairs of indicators) rather than leveraging information to estimate the parameters of interest. On the other hand, SEM is more parsimonious; the information was efficiently used to estimate the parameters of interest. This comparison highlights the potential advantage of modeling common underlying mechanisms by using SEM when there are such mechanisms.
